# Surgical inflammatory stress: the embryo takes hold of the reins again

**DOI:** 10.1186/1742-4682-10-6

**Published:** 2013-02-01

**Authors:** Maria-Angeles Aller, Jose-Ignacio Arias, Isabel Prieto, Carlos Gilsanz, Ana Arias, Heping Yang, Jaime Arias

**Affiliations:** 1 Department of Surgery, School of Medicine, Complutense University of Madrid, Madrid, Spain; 2 General and Digestive Surgery Unit, Monte Naranco Hospital, Oviedo, Asturias, Spain; 3 Department of General and Digestive Surgery, La Paz Hospital, Autonomous University, Madrid, Spain; 4 General and Digestive Surgery Unit, Sudeste University Hospital, Arganda del Rey, Madrid, Spain; 5 Department of Medicine, Puerta de Hierro Hospital, Autonomous University, Madrid, Spain; 6 Division of Gastroenterology and Liver Disease, USC Research Centre for Liver Diseases, Los Angeles, CA, USA

**Keywords:** Surgical inflammation, Stress, Wound healing, Amniotic, Yolk sac, Vitelline, Gastrulation

## Abstract

The surgical inflammatory response can be a type of high-grade acute stress response associated with an increasingly complex trophic functional system for using oxygen. This systemic neuro-immune-endocrine response seems to induce the re-expression of 2 extraembryonic-like functional axes, i.e. coelomic-amniotic and trophoblastic-yolk-sac-related, within injured tissues and organs, thus favoring their re-development. Accordingly, through the up-regulation of two systemic inflammatory phenotypes, i.e. neurogenic and immune-related, a gestational-like response using embryonic functions would be induced in the patient’s injured tissues and organs, which would therefore result in their repair. Here we establish a comparison between the pathophysiological mechanisms that are produced during the inflammatory response and the physiological mechanisms that are expressed during early embryonic development. In this way, surgical inflammation could be a high-grade stress response whose pathophysiological mechanisms would be based on the recapitulation of ontogenic and phylogenetic-related functions. Thus, the ultimate objective of surgical inflammation, as a gestational process, is creating new tissues/organs for repairing the injured ones. Since surgical inflammation and early embryonic development share common production mechanisms, the factors that hamper the wound healing reaction in surgical patients could be similar to those that impair the gestational process.

## Introduction

Hans Selye described the stress response or General Adaptation Syndrome as a non-specific response to any stressor that occurs in three stages: general alarm reaction, resistance and exhaustion [1]. This syndrome was compared by Selye to other general defense reactions, such as the inflammation syndrome, particularly when secondary to surgical injury [1,2].

The considerable progress that has been made in discerning the cellular and molecular mechanisms of the stress-response [3-9] and the inflammatory response [10-15] enables establishing close links between both types of defense reactions [16-21]. All the new biochemical knowledge could also allow for a comprehensive interpretation of the complex interactions between the nervous, immune and endocrine responses triggered by a stressor during the evolution of both general reactions, and most especially when related to surgery.

## Surgical inflammation

The inflammatory response related to surgery, either elective or anesthetized injury, or trauma-related, i.e. accidental or anaesthetized injury, could be a surgical inflammation [14]. Surgical inflammation usually associated with psychological stimuli occurs with pathophysiological changes associated with the stress response [22], which is why the stress response could have evolved from and is intricately linked to the surgical inflammatory response [16,22]. Thus, another appropriate name for this phenomenon is trauma-induced inflammatory stress.

From a general point of view, surgical inflammation is a systemic response of the body. Surgical-related inflammation varies in intensity, depending on the severity of the injury suffered. The pathophysiological characteristics of this systemic response are obvious in the cases of severe injury, as seen in polytraumatized patients [14,23-25]. In the cases of mild injury, however, such as in skin wound healing, the systemic response is imperceptible or subtle [22].

Severe injury induces a clear systemic inflammatory response in the body that appears to develop through the expression of 3 successive and overlapping phenotypes: the neurogenic, immune and endocrine [12,26] (Table 1). The mechanisms that control the systemic surgical inflammatory response would be based on the increasing metabolic ability of the body to use oxygen over the successive phases of its evolution towards the tissue repair. The metabolic ability of each phenotype would in turn be determined by the mechanisms used for cellular energy production [12,26].

**Table 1:** Surgical inflammation could be viewed as a high-degree stress response of the patient composed of three overlapping successive phenotypes

**INFLAMMATORY PHENOTYPES IN SURGICAL INFLAMMATION**		
**NEUROGENIC**	**IMMUNE**	**ENDOCRINE**
- Stress sensation	- Bone marrow-related response	- Epiblast-derived pluripotent stem cells
- Inflammatory pain	- Hematopoietic stem cell activation	- Mesenchymal stem cell activation
- Fight-to-flight effect	- Signaling molecules:	- Fibrocytes
- Analgesia	* Chemokines	-Endothelial progenitor cells
- Hypothalamic-pituitary-adrenal cortical activation	* Toll-like receptors	- Signaling molecules:
	* Cytokines	* Chemokines and their receptors
- Sympatico-adrenal medullary activation	- Leukocyte activation	- Anabolic hormones
- Tachycardia	- Bacterial translocation	* Insulin
- Shock	- Enzymatic stress	* GH
- Ischemia-reperfusion	- Acute phase response	* IGF1
- Renin-angiotensin-aldosterone axis activation	- Coagulaton/Complement system activation	- CARS
		- Resolution
- Hydroelectrolytic alterations	- Coagulopathy	* Lipoxin
- Interstitial edema	- Dyslipidemia	* Resolvins
- Increased lymph flow	- SIRS/MODS	* Protectins
- Hypoxia	- Lymph node activation	- Angiogenesis/vasculogenesis
- Anaerobic acidosis	- Local stress response by leukocytes	- Blood capillaries
- Hypothermia	- Local cholesterol-derived hormones	- Specialezed epithelium
	- Hypercatabolism	- Energetic stress
	- Hypermetabolism	- Oxidative phosphorylation
		- Centralization of neuroendocrine functions
		- PTSD

CARS: Compensatory Anti-inflammatory Response Syndrome. SIRS/MODS: Systemic Inflammatory Response Syndrome/Multiple Organ Dysfunction Syndrome. PTSD: Post-Traumatic Stress Disorder.

**Figure 1:**
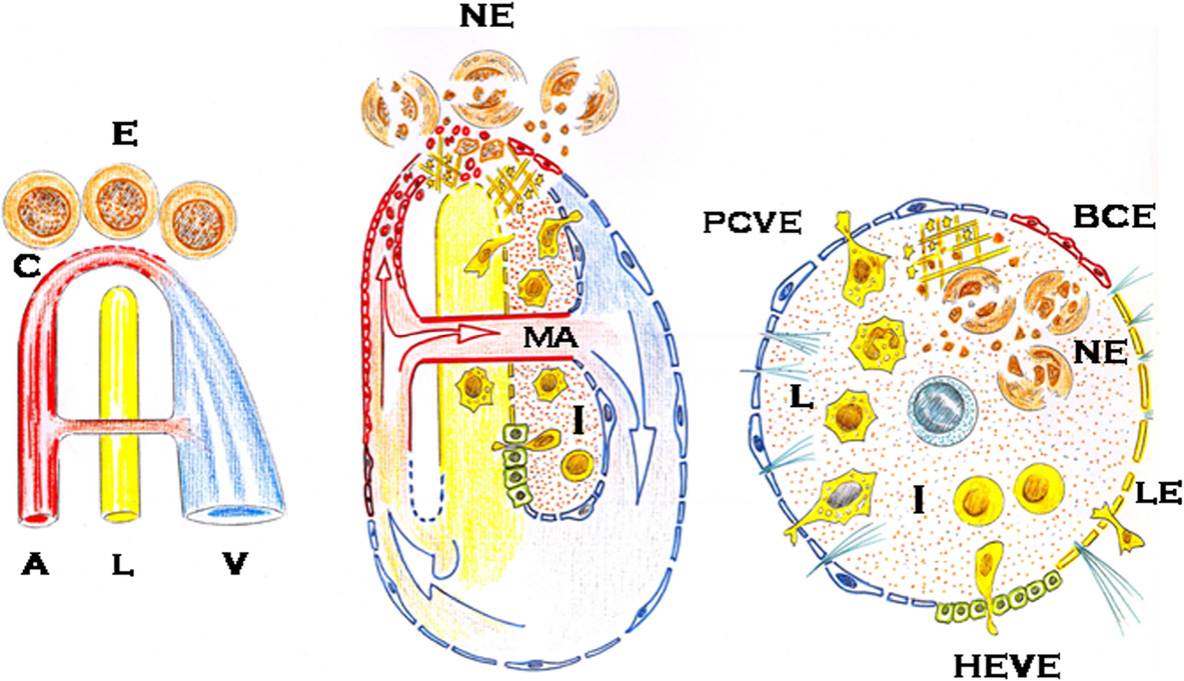
**Schematic representation of microcirculation.** In a steady state, the arterial blood flow moves through the capillaries, where it’s deoxygenated before reaching the post-capillary venule. The lymphatic drainage from the interstitial space is reduced and there is a dynamic balance between the formation and the elimination of the interstitial fluid (left). When inflammation is produced, the arterial blood flow can’t be deoxygenated, firstly because the epithelium is necrosed and, secondly because the capillaries are also necrosed and/or obstructed. Arterial blood flow bypass through metaarteriolas prevents the post-capillary vein from being exposed to high oxygen levels. In addition, lymphatic flow gains an unusual prominence (middle). The inflamed interstitium could be represented as surrounded by the different types of endothelium that make up microcirculation, as an “endothelial egg”. The venous and lymphatic endothelium acquire higher extension and functionality since the molecular and cellular exchange between the inflamed interstitium and the rest of the body, that is the host, is produced through them. A: artery; BCE: blood capillary endothelium; C: capillary; E: epithelium; HEVE: high endothelial venule endothelium; I: interstitium; L: lymphatic; LE: lymphatic endothelium; Leu: leukocytes; MA: meta-arteriole; NE: necrosed epithelium; PCVE: postcapillary venule endothelium; SC: stem cell.

The above-mentioned phenotypes, which characterize the evolution of the systemic inflammatory response to the injury, have been represented as focused and integrated within an interstitial circular space restricted by the different types of the endothelium making up the microcirculatory system (Figure 1). This suggests that the systemic endothelial dysfunction, in its broadest sense, not only affects the three principal types of endothelium that are involved in the inflammatory response – the blood capillary, lymphatic and venous-, but also produces an endothelial-type redistribution with the progressive prominence of the venous and lymphatic endothelium at the expense of the blood capillary endothelium. When the systemic endothelium functions normally, the blood capillary endothelium would therefore recover its leading role mediating the exchange of oxygen, nutrients and waste products. Meanwhile, the inflammatory activity mediated by the venous and lymphatic endothelium would be dramatically reduced [26] (Figure 1). The molecular and cellular mediators of the host stress response to the injury are therefore selected for passing through the heterogeneous endothelial barrier that limits the interstitium, where the inflammatory battle will be fought.

## The neurogenic inflammatory phenotype

**Figure 2:**
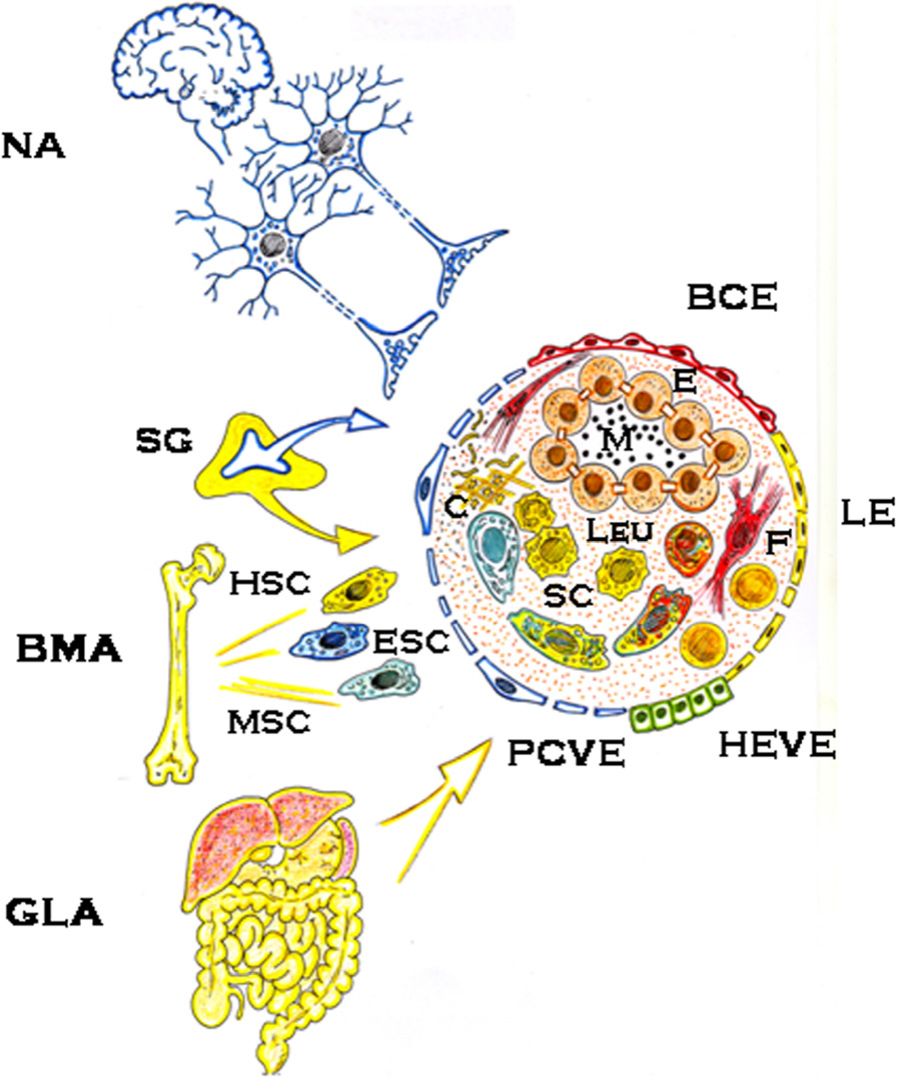
**Surgical stressful inflammatory response.** The host organism provides the inflamed interstitium molecules and cells by up-regulating a neurogenic-related axis (NA), a bone marrow-related axis (BMA) and a gut-liver axis (GLA). The molecular and cellular infiltration of the “endothelial egg” induces the development of a new tissue or organ using inflammatory mechanisms. BCE: blood capillary endothelium; C: coagulation; E: epithelium; ESC: endothelial precursor cell; F: fibroblast; HEVE: high endothelial venule endothelium; HSC: hemopoietic stem cell; LE: lymphatic endothelium; Leu: leukocytes; M: microbiome; MSC: mesenchymal stem cell; PCVE: postcapillary venule endothelium; SC: resident stem cell; SG: suprarenal gland.

The systemic inflammatory response begins with an immediate pathological neuromuscular response that includes sensitive impairments like stress sensation, inflammatory pain, analgesia and motor alterations. In addition, skeletal muscle is also involved, i.e. the fight-to-fight effect, behavior and withdrawal reflexes, the myocardium-tachycardia- and the vascular smooth muscle with vasoconstriction and vasodilatation, which induces systemic and local hemodynamic impairments, i.e. blood flow redistribution and ischemia-reperfusion [25,26]. A common and basic pathogenic mechanism of this complex neuromuscular response would be sudden hydroelectrolytic alterations [26]. Consequently, there is increasing evidence that the systemic inflammatory response is actually associated with abnormal ion transport [27]. In this phase of surgical inflammation, interstitial hydroelectrolytic alterations therefore stand out and nutrition by diffusion predominates. The ischemia-reperfusion phenomenon, which causes oxidative and nitrosative stress, could be responsible for exudation and the progression of the interstitial edema. While edema is being produced, the lymphatic circulation is activated [22,25]. Since the inflammatory interstitium is initially hypoxic and shows metabolic anaerobic acidosis, mainly due to the accumulation of acidic by-products including lactate, the hypoxic environment could represent an ideal stem cell niche [28,29] (Figure 2).

In the early evolutionary period of the neurogenic stress response, the hypothalamic-pituitary-adrenocortical, sympathetic-adrenal medullary and renin-angiotensin-aldosterone axes, with the secretion of catecholamines, glucocorticoids and mineralocorticoids in the circulation, are activated [5,30-33]. Chromaffin vesicles in adrenal medullary chromaffin cells also store granins, which can function as prohormones giving rise to bioactive peptides, some with potent antimicrobial activity [34]. Consequently, these substances are selectively accumulated in the interstitial space of the tissues suffering from ischemia-reperfusion because endothelial permeability is increased, especially in the post-capillary venules [12,22,25]. The elevated central venous saturation of oxygen seen in many patients with multiple organ dysfunction may therefore be driven by the hyperdynamic state with shunting through the microcirculation and/or cellular dysoxia [35] (Figure 2).

## The immune inflammatory phenotype

The immune phenotype is the intermediate phase of the systemic inflammatory response to the stressor. In this phase, the tissues and epithelial organs, which have previously suffered damage by ischemia-reperfusion, are infiltrated by inflammatory cells and bacteria. This infiltration occurs in an edematous oxygen-poor environment [22,25]. Today, the inflammatory bone-marrow-related response is considered both a key and complementary arm of the stress response [22]. The inflammatory activation of the bone marrow stem cell niche indicates the stimulation of the hematopoietic stem cells (HSCs) and the mesenchymal stem cells (MSCs), both multipotent stem cells, [36]. HSCs are the progenitors of all blood and immune cells that infiltrate all the tissues and organs that have been previously primed by oxidative and nitrosative stress. Inflammatory signaling molecules, including interferons, tumor necrosis factor-alpha and toll-like receptors, appear to stimulate HSC proliferation in the short term [37]. In turn, interferon-gamma mediates HSC stimulation in response to chronic inflammation [38]. This immune phase represents enzymatic stress with processes related to intracellular digestion, i.e. autophagy, phagocytosis and antigen presentation, and extracellular digestion, i.e. fermentation, all of which favor tissue trophism [12,26]. Macrophages and dendritic cells also take advantage of the lymphatic circulation activation. Macrophages migrate within the lymphatic circulation until reaching the lymph nodes where they activate lymphocytes [26,39] (Figure 2).

The cells that infiltrate the interstitium in the inflamed tissues and organs, thanks to the open microcirculatory system, acquire metabolic characteristics that transform them into tissues with great functional autonomy. Thus, monocytes, macrophages and lymphoid cells express β- and α_1_-adrenoceptors and catecholamines [40-42]. Lymphocytes store and synthesize serotonin [43] and immune cell-derived cholinergic activity can modulate inflammation [44,45]. Furthermore, the pro-opiomelanocortin (POMC)-peptides found in leukocytes, including adrenocorticotrophin (ACTH), exhibit the same structure of those present in the pituitary [46,47]. POMC-derived peptides have a very ancient origin and an extremely high level of conservation [48]. They also play a critical role in regulating energy balance [49]. Maybe these are the reasons why the stress response axis can be retrieved by the immunocytes participating in the inflamed interstitium of tissues and organs, where the corticotropin-releasing hormone (CRH), ACTH-like and biogenic amines are present [47].

In this phase of the inflammatory response, the acute phase reaction becomes more prominent and exhibits diverse pathophysiological changes such as pyrexia, leukocytosis and dramatic changes in the plasmatic concentrations of acute phase proteins [50,51]. Acute phase proteins are circulating biomarkers of inflammation and are defined as either positive or negative, depending on whether they increase or decrease during the inflammatory response [50,52]. Negative acute phase proteins include albumin, transferrin and retinol-binding protein [51]. Positive acute phase proteins are synthesized by hepatocytes in response to IL-6 as part of the innate immune response [53]. Skeletal muscle proteolysis is suggested as the main source for free amino acids for the hepatic acute phase response [54]. Positive acute phase proteins include proteins of the coagulation-fibrinolysis system (fibrinogen, prothrombin, factor VIII, von-Willebrand factor, complement factors, plasminogen), protease inhibitors (alpha-1-antitrypsin, alpha-1-antichymotrypsin), transport proteins (ceruloplasmin, hemopexin, haptoglobin) and lipid transport proteins (serum amyloid A and serum amyloid P) [50,51]. However, C-reactive protein is the main human acute phase protein and one of the most sensitive markers of inflammation, which is why biochemical tests are frequently requested [50,55].

Trauma-induced coagulopathy is a biphasic process. Coagulopathy is initially related to excessive hyperfibrinolysis, hyperfibrinogenolysis, hypothermia, acidosis and hypometabolism. These changes affect the coagulation system and produce inappropriate anticoagulation with hemorrhagic tendency [56]. Then, the acute post-traumatic stress correlates with procoagulant changes, such as increased levels of fibrinogen, clotting factors VII, VIII and XII and the von Willebrand factor [57]. Consequently, those patients who develop sepsis can suffer disseminated intravascular coagulation, with intravascular microcoagulation and subsequent consumption coagulopathy [56,58].

Adrenal medullary epinephrine secretion exhibits robust hepatic glycogenolysis that rapidly elevates blood glucose concentrations, but also simultaneously stimulates lipolysis [59]. The major alterations in lipid metabolism during the systemic inflammatory response syndrome include increased lipolysis and fatty acid recycling, hypertriglyceridemia and hepatic steatosis. Hypocholesterolemia, however, is common in patients with critical illness and may be valuable for prognosis [58]. Serum amyloide A increases the ability of HDL to serve as an acceptor for cellular cholesterol efflux, promoting the removal of excess cholesterol from macrophages as well as increasing the availability of cellular free cholesterol [60]. It has been suggested that acute-phase serum amyloid A is part of a systemic response to injury to recycle and reuse cholesterol from destroyed and damaged cells. In this case, the recycling of cholesterol during serious injury could play an important survival role [61]. In fact, the predominance of the lipid metabolism with accumulation of cholesterol in the inflamed tissue could be attributed to its role as a precursor molecule of many hormones, including aldosterone, corticoids, progesterone, androgens and strogen [62], and even vitamin D [63].

Major injury due to surgical or major trauma produces potentially severe immunological dysfunction resulting in the systemic inflammatory response syndrome (SIRS) and early multiple organ dysfunction syndrome (MODS), mediated primarily by the innate immune system [23-25,64]. This is followed by a compensatory anti-inflammatory response syndrome (CARS) and late MODS, which are primarily mediated by the adaptive immune system [23,24,64]. Infection and sepsis may exhibit organ system dysfunction rather than its cause [65]. If so, gastrointestinal dysfunction and associated microbiome dysfunction may cause bacterial intestinal translocation to colonize the inflamed interstitium in the sickest patients with immunoparalysis [25,65]. Hypercatabolism and hypermetabolism are both characteristic metabolic alterations of SIRS after polytrauma [24]. This metabolic association could suggest that, during the evolution of the immune response due to a severe injury, the body suffers a deconstruction process. The body would then obtain the substrates it needs to gestate new tissues and organs for self-repair.

## The endocrine inflammatory phenotype

Nutrition mediated by blood capillaries is established by angiogenesis. The new functional properties of the microcirculation include the exchange of oxygen, nutrients and waste products. This oxygen support induces oxidative metabolism. This is an efficient method for extracting energy from blood molecules, which begins with the Krebs cycle and ends with oxidative phosphorylation [12,25]. Oxygen and oxidative metabolism are an excellent combination through which cells can obtain an abundant energy supply for tissue and organ repair using specialized cells, although this combination also produces energetic stress [25].

Little is known, however, about the capacity of eukaryotic cells to monitor the redox state for supporting specialized functions [66]. Although NF-κB mainly triggers inflammation, it also resolves it [67]. Clearly, the mechanisms that promote tissue structuring and function restoration also include those involved in resolving inflammation [68]. In endogenous pro-resolving lipid mediators, lipoxins, resolvins and protectins are the most studied. In essence, pro-resolving factors return the pro-inflammatory phenotype to its prior physiological state and therefore the microcirculatory functions of tissues and organs return to homeostasis [69].

Inflammatory chemokines trigger the interstitial recruitment of leukocytes, but also act as a signal for homing endothelial progenitor cells from the bone marrow to the sites of angiogenesis [70]. A novel mechanism has been identified in vasculature development known as vasculogenesis, or new vessel. These new vessels form from bone marrow derived endothelial progenitor cells [71] rather than from the sprouting or elongation of existing vessels [72,73]. During vasculogenesis or neovascularization, precursor cells from adult bone marrow are mobilized into circulation in response to various signals, including chemokines and their receptors. They are also homed to the source where they differentiate into mature endothelial cells [74]. Although the major physiological role of circulating endothelial progenitor cells in adults is to ultimately maintain vascular integrity, they also home to and aid to revascularize ischemic inflammatory organs and tissues [71]. In repair, however, mesodermal-derived cells, particularly fibrocytes derived from the bone marrow stem cell niche, like MSC [75] or, alternatively epiblast-derived pluripotent stem cells that survive beyond embryonic development in adult tissues as so-called “hibernating backup” [76], play the leading role in the repair reaction. Fibrocytes can accelerate the recovery of homeostasis by the injured organism through stimulating proliferation and vessel remodeling. The recruitment of mural mesenchymal derived cells, i.e. pericytes and vascular smooth muscle cells, to nascent blood vessels, plays an important role in stabilizing and maturing new vascular networks [72,73].

Nutrition mediated by mature blood capillaries is established through the expression of this angiogenic and/or vasculogenic phenotype, which makes tissue and organ regeneration possible or, by default, their repair through fibrosis [22,25].

In this convalescence phase, the hypercatabolic syndrome is progressively down-regulated with the reduction of catabolic hormones and/or molecules, i.e. catecholamines, pro-inflammatory cytokines, cortisol and glucagon and the increase of anabolic hormones, i.e. insulin, growth hormones, insulin-like growth factor-1, or anabolic steroids, which are supported by tissues and organs through the new vessel arrangement and morphology [77].

Since the above-mentioned neurogenic and immune phenotypes become autonomous when expressed in the peripheral inflamed area, during the last endocrine phenotype expression, the body regains control over the neoformed tissue. This progressive centralization of the neuroendocrine functions, i.e. sympathetic and vagal nervous system- and endocrine- hypothalamic-pituitary-organ-hormonal axes, could be related to the progressive remodeling of the tissues and organs, controlled in turn by hemodynamic and metabolic stimuli [78]. The failure to correctly centralize the neuroendocrine axes, however, would cause a chronic dysregulation with dysfunctional impairment and patients would suffer post-traumatic stress disorders in the long term [79,80].

## The inflammatory *interstitium* behaves like an endothelial egg

**Figure 3:**
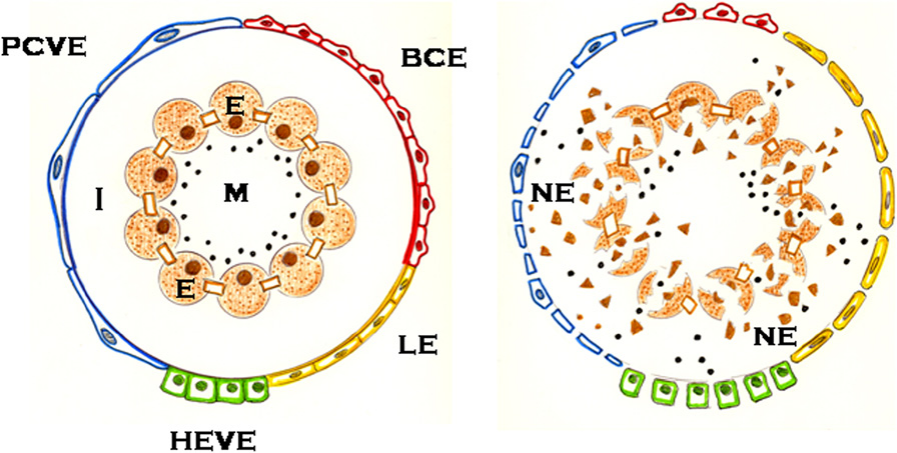
**Schematic representation of normal tissue (left) and pathological (right) tissue when surgical inflammatory stress is developed.** Epithelial necrosis triggers an inflammatory response in the interstitium that is restricted by an endothelial barrier made up of the different types of microcirculatory endothelium. BCE: blood capillary endothelium; E: epithelium; HEVE: high endothelial venule endothelium; I:interstitium; LE: lymphatic endothelium; M: microbiome; NE: necrosed epithelium; PCVE: post-capillary venule endothelium.

The interstitium is considered as the space where the battle of inflammation develops. In particular, during the surgical inflammatory response, the interstitial space increases its size exceedingly as a consequence of the successive infiltration it suffers by molecules, cells and bacteria. Since these molecular, cellular and bacterial inflammatory mediators enter the interstitium through an endothelial pathway, the interstitial inflammatory space could be represented as an “endothelial egg”. Thus, this inflammatory endothelial egg would get cellular and bacterial substrates through the postcapillary venule endothelium, the high endothelial venule endothelium in the lymph nodes and, in lesser degree, through the capillary endothelium. The lymphatic endothelium could therefore be equivalent to an excretory system of the endothelial inflammatory egg (Figure 3). Consequently, the successive phases of the inflammatory response, which ultimately would lead to the production of a new tissue formed by parenchyma and stroma, would be produced in the interior of this hypothetical endothelial egg.

However, if harmful factors have an impact during the normal development of this hypothetical inflammatory egg, formation of a tissue similar to a pre-existing one by regeneration would be hindered, so the stromal component of the tissue would predominate and fibrosis would be induced. The deficit or excess of the above-mentioned molecular, cellular or bacterial mediator support also impairs the normal development of the inflammatory egg since it would prevent the development of a new tissue. Consequently, the inflammatory response would be chronic and finally, healing would fail.

In surgical-related inflammation the *interstitium* is surrounded by a heterogenous endothelium. The different types of microcirculatory vascular endothelium that surround the inflammatory interstitium could function like an endothelial thermostat that autoregulates bodily functions with the ability to return to normal in the short-term. However, when the autoregulating ability of the interstitium is defective, an endothelial dysregulation inducing a chronic inflammatory pathology would be produced [26]. If we focus on the generic representation of the inflamed tissues and organs as an “endothelial egg”, several hypothetical evolutive proposals could be considered during this egg development. The different types of evolution could range between its normal development and hitching, thanks to the influx of the mentioned inflammatory phenotypes induced by the host and the defective development due to noxious factors, which would involve endothelial egg development (intrinsic factors) and an effective response (by the host) to stress that induces normal gestation (extrinsic factors) (Figure 3).

## Recapitulated extraembryonic functions related to surgical inflammatory phenotypes

Surgical-related inflammation could recapitulate ontogeny by re-expressing 2 hypothetical extraembryonic trophic axes (amniotic and yolk sac or vitelline) in the interstitial space of the injured tissue [22,78]. If so, surgical –related inflammation could represent the postnatal debut of ancestral biochemical mechanisms that were used for normal embryonic development. The re-expression of these ancient mechanisms is perhaps hard to recognize, since they are anachronistic during postnatal life and are established in a different environmental medium [22].

**Figure 4:**
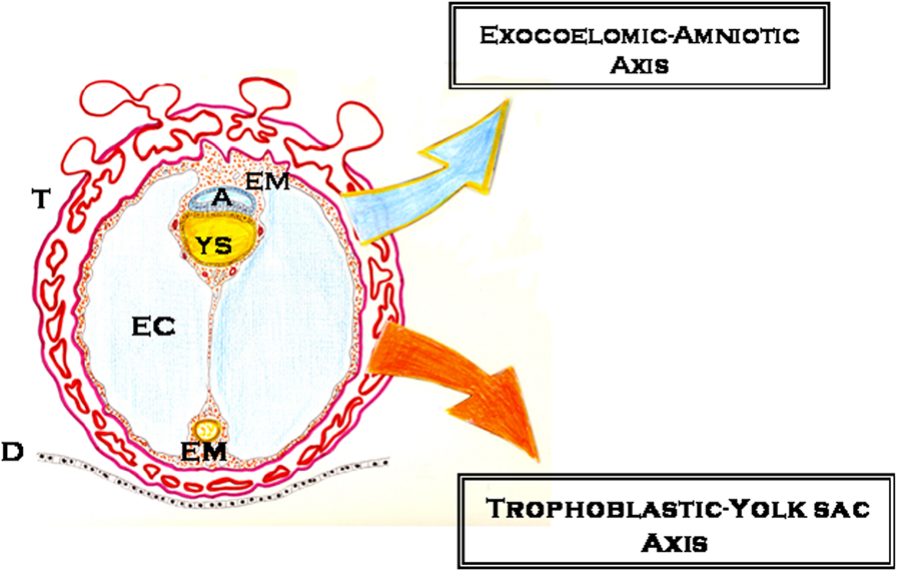
**Schematic representation of early embryonic development.** During early embryo development, the extraembryonic mesoderm and the exocoelomic cavity (EC) relate the trophoblast (T) to the amnion and the secondary yolk sac (YS). The existence of two extra-embryonic axes, an exocoelomic-amniotic axis and a trophoblastic-yolk sac axis could be proposed. This would enable the formation of the intraembryonic mesenchymal since they are integrated. These extraembryonic functions are expressed by the host organism when it suffers an injury and focuses on the injured tissue or organ. After these functions are incorporated by the injured tissue or organ, this said tissue or organ acquires the embryonic functional autonomy needed to successfully repair itself. A: amnion; EC: exocoelomic cavity; EM: extraembryonic mesoderm; D: decidua; T: trophoblast; YS: secondary yolk sac.

After fertilization, the first stage of embryogenesis is the zygote, which undergoes cleavage by mitosis. When the morula stage is reached, the embryo establishes polarity. The cells bind tightly to each other, forming a compact sphere (or blastocyst) with 2 different cell layers. The outermost layer becomes the trophoblast, giving rise to the placenta. The inner cells become the inner cell mass, giving rise to the embryo and the remaining structures, including the amnion, yolk sac, and allantoids [81]. The extraembryonic *coeloum* or exocoelomic cavity surrounds the blastocyst, which is composed of two structures, the amnion and the primary yolk sac. At the end of the fourth week of gestation, the developing exocoelomic cavity splits the extraembryonic mesoderm into two layers, the somatic mesoderm, lining the trophoblast, and the splanchnic mesoderm, covering the secondary yolk sac and the embryo [82] (Figure 4). Coelomic fluid results from an ultrafiltrate of maternal serum with the addition of specific placental and secondary yolk sac by-products [82,83].

The hypothetical recapitulation of these initial phases of the embryonic development during the early surgical inflammatory response would imply the expression of functions similar to the extraembryonic coelom that surrounds the blastocyst. Accordingly, the phenotype could be adopted by the inflamed interstitium that subsequently induces the accumulation of fluid with similar characteristics to coelomic fluid in an environment with low pH and oxygen [82-84]. In essence, interstitial edema with high levels of proteins, in particular albumin, as well as electrolytes, metals, amino acids, antioxidants, cytokines and cholesterol-derived hormones would be produced in the inflammatory exudate [82-88]. This initial interstitial pro-inflammatory edema secondary to ischemia-reperfusion would play the leading role in the trophism of the injured tissue [12,89]. Biological data and the anatomical location of the exocoelomic cavity also suggest that it plays a major role as an essential nutritional pathway for the embryo and early fetus before placental circulation is fully established [83].

The amnion is an embryonic functional axis with strong neural potential [90]. Amnion-derived multipotent progenitor cells secrete a unique combination of cytokines and growth factors, called the “amnion-derived cellular cytokine solution”, which establishes a connection between mesenchymal and epithelial cells during embryo development [91]. The amniotic fluid surrounding the fetus may therefore be an extension of the extracellular space of the fetal tissues [92]. Furthermore, pluripotent stem cells within the amniotic fluid could be a new source for stem cell research [93,94]. The amniotic-like phenotype could offer the stem cell a hypoxic and hydrated interstitial axis [92] with cytokines and growth factors [91] favoring not only nutrition by diffusion, but also transport, excretion and bacteriostatic and anti-inflammatory protection [92,94] (Figure 4).

The wall of the secondary yolk sac in mammals is formed by an external mesothelial layer facing the exocoelomic cavity, a vascular mesenchyme and an endodermal layer facing the yolk sac cavity [82] (Figure 4). The formation of blood islands in the mesenchymal layer promotes the development of hematopoiesis and angiogenesis [95]. The endothelial and blood progenitors of blood islands develop independently from the epiblast before they migrate to the extraembryonic yolk sac. Hemangioblasts found in these blood islands could generate blood cells through intermediate progenitors called hemogenic endothelial cells [96]. From the sixth week of gestation, the secondary yolk sac appears as a cystic structure covered by numerous superficial small vessels [82]. The mesothelial and endodermal layers have absorptive functions and are active in endocytosis/digestion [95,97]. In addition, the endodermal layer is the source of several proteins including acute phase proteins, such as prealbumin, albumin, transferrin and α_1_-antitrypsin [93], as well as α-fetoprotein, which is produced by both the adult and fetal liver [82,96].

A major function of the yolk sac is carbohydrate, protein and lipid accumulation for embryo nutrition (*vitellum*) [98]. The yolk sac therefore provides lipids and lipid-soluble nutrients to embryos during the early phases of development [98]. Particularly, the yolk sac endoderm is involved in the regulation of vascular integrity of developing embryos [99]. During the 10^th^ week of gestation, however, the yolk sac starts to degenerate and rapidly ceases to function [82]. The yolk sac-related phenotype could favor the regulation of lipid metabolism genes [99], the hematopoietic-cell derived control with recruitment of immune cells and the induction of an angiogenic switch [96] to enable new tissue immunological tolerance during the surgical inflammatory response. In addition, through the synthesis and release of acute phase proteins, this extraembryonic phenotype reduces oxidative, nitrosative and enzymatic stress and activates the complement-coagulation system, regulates the lipid metabolism [50,51] and favors phagocytosis [51], a specific form of endocytosis primarily associated with nutrition in unicellular organisms i.e. phagotrophic nutrition, and with innate and adaptive immunity in mammals [100].

During trophoblast differentiation, trophoblast cells also exhibit intense phagocytic activity leading to events as diverse as engulfment and destruction of extracellular material and the production of inflammatory mediators that may modulate both the immune response [100] and trophoblast invasiveness [101,102].

## The recapitulated amniotic-vitelline axes and the interstitial inflammatory endothelial egg

The molecular and cellular contribution made by the above-mentioned extraembryonic membranes, i.e. exocoelomic cavity, amnion, yolk sac and trophoblast to the intraembryonic mesoderm, could be essential for embryo development and organogenesis [78]. Moreover, these primitive extraembryonic structures can be internalized by the embryo at early developmental stages [103]. Consequently, the hypothesized re-expression of these extraembryonic functions after injury during postnatal life could be a key process needed to repair the injured organism [22,78].

In this way, both the amniotic and vitelline axes re-expressed during the interstitial inflammation would contribute, to the “interstitial inflammatory endothelial egg”, those molecules and cells that would be necessary during evolution of the wound healing response to form a new tissue by regeneration and/or by scarring. Therefore, these two axes act on the inflamed interstitium in a similar fashion as they act during embryonic development, although in this case an embryo is not created, but the injured tissue is repaired using similar mechanisms.

**Figure 5:**
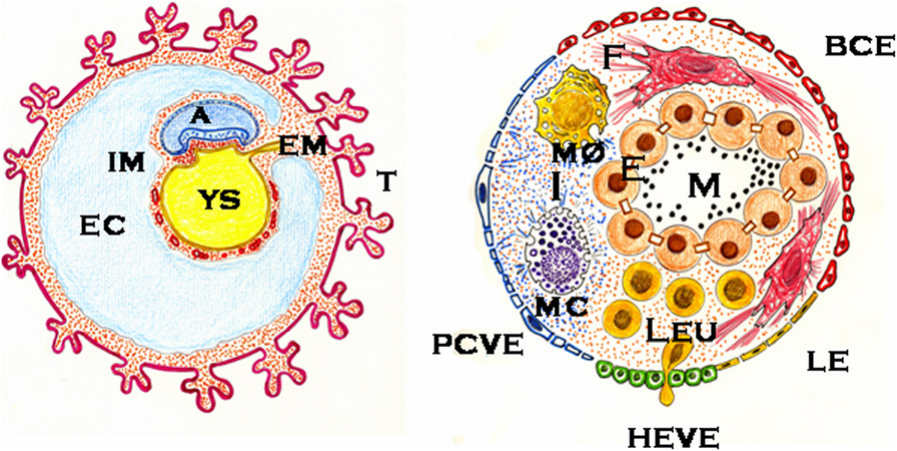
**Comparative representation between the embryo, with its extra-embryonic membranes (left) and the inflamed tissue (right).** Recapitulation of the extra-embryonic exocoelomic-amniotic (E-A)- axis and trophoblastic-yolk sac-related (T-YS) axis within the inflamed tissue would allow the development of a new tissue from the intra-embryonic mesoderm, as it occurs during embryonic development. BCE: blood capillary endothelium; E: epithelium; EC: exocoelomic cavity; EM: extra-embryonicmesoderm; F: fibroblast; HEVE: high endothelial venule endothelium; IM: intraembryonic mesenchyma; LE: lymphatic endothelium; Leu: leukocyte; M: microbiome; MC: mast cell; MØ: Macrophage; PCVE: postcapillary venule endothelium; YS: yolk sac.

The representation of the inflamed interstitial tissue like a sphere whose surface is covered by different types of endothelium -arterial capillary, venous and lymphatic- allows for a better understanding of the specific function of these endothelia in relationship with the embryo-like metabolism that is developed in this interstitium. The type of substrates and cells or bacteria that the new developing tissue needs in each evolutionary phase of inflammation could be modified by changing the venous incretory phenotype and the metabolism waste products. This aim can be achieved by using the different excretory functions of the lymphatic endothelium that surrounds the new formed tissue (Figure 5).

## Coupling the re-expressed extraembryonic phenotypes with postnatal survival

The systemic surgical-related inflammatory response and, therefore trauma-induced systemic inflammatory stress is a complex process involving systemic alterations related to a stress response. The magnitude of this systemic response, however, could reflect the demands required for tissue and organ repair. Here we have been trying to establish similarities between the complex pathophysiological mechanisms developed in surgical inflammation and the pluripotential extraembryonic pathways during embryo development. During the evolution of the surgical inflammatory response, the re-expressed extraembryonic functions target the injured tissues and this would condition the evolution of the repair response. Embryonic programs adapted to the severity of the injury would be then be activated to begin repair. The recapitulation of the extraembryonic coelomic and amniotic functions could be represented by initially activating the systemic neurogenic axis, while the latter recapitulation of the trophoblast and yolk sac functions would be carried out by activating a systemic bone-marrow-related axis.

The activation of the pathological systemic axes, i.e. neurogenic and bone marrow-related, in the injured tissue could be completed in 2 steps. First, the upregulated amniotic phenotype would induce a sudden and early neurogenic response with systemic cardiovascular, hemodynamic, and hydroelectrolytic alterations. In this early response, cells that produce substances for export first synthesize and then store large amounts of molecules, such as biogenic amines [22], in secretory vesicles ready for rapid release [104]. In turn, the upregulated systemic vitelline phenotype could mediate the inflammatory bone marrow response through a lipid metabolic switch linked to steroid and acute phase response protein synthesis, respectively [22]. This slower response would therefore be developed by steroidogenic cells that store very little steroid hormones, in which case a rapid steroidogenic response would require immediate synthesis of new steroids, such as cortisol. The increase of the acute-phase protein synthesis, i.e. innate immunity, by the hepatocytes is linked with the acute phase response and follows the upregulation of pro-inflammatory cytokines and chemokines [50,51].

Finally, the systemic pathological axes, i.e. neurogenic and bone-marrow related, are focused and coupled in the interstitium of the injured tissues and organs. This interstitial integration of both pathological axes in the injured tissues and organs could finally induce a gastrulation-like process [78]. Gastrulation, which involves the de novo formation of reparative tissue, is based on the recapitulation of the intra-embryonic mesenchyme formation process [78,81]. In essence, the integration of both extraembryonic-related phenotypes, coelomic-amniotic and trophoblast-vitelline, by the multipotent mesenchymal stem/stromal cells [105-107] would support the functional and metabolic heterogeneity needed to successively modulate their injured microenvironment during the evolution of the surgical inflammatory response [105,107]. Therefore, the interaction of extraembryonic functional axes in the interstitium of the damaged tissue and organs allows for the recapitulation of the mechanisms characteristic of gastrulation, subsequently forming a mesenchyme similar to that present in the early development phases [22,78].

Mesodermal-derived cells, particularly fibrocytes [108] or alternatively, epiblast-derived pluripotent stem cells that survive beyond embryonic development in adult tissues and known as the “hibernating backup” [109], play the leading role in the repair reaction. Fibrocytes are bone marrow-derived mesenchymal progenitors that express surface markers for leukocytes and mesenchymal cells [110]. In addition, fibrocytes can accelerate wound healing by stimulating cell proliferation, reepithelialization, and angiogenesis [111], although they can also be involved in inflammatory fibrotic processes [112,113].

## A phylogenetic point-of-view about surgical inflammation

Since it has been hypothesized that during the surgical-related inflammatory response, extraembryonic axes i.e. coelomic-amniotic and trophoblastic-yolk sac-related, are systemically re-expressed and converge into the interstitium of injured tissues and organs, the final aim of this process could also be collaborating in some type of development similar to embryonic development during the postnatal life [22].

The repair process could therefore have properties comparable to an embryo. One of the main objectives of the upregulated extraembryonic axes could be collaborating in the establishment of an open circulation in the injured tissues and organs during the early phase of the inflammatory response. An open circulatory system could facilitate interstitial fluid diffusion and concomitantly tissue nutrition when the blood circulation loses its prominent role [22,114]. Animals have adapted their circulatory system during phylogeny. Vertebrates have a closed circulatory system and a heart that drives blood through closed blood vessels [115], whereas in invertebrates, like insects, the body fluid (hemolymph) is pumped by a dorsal heart through the tissues with no closed vessels [116]. In the inflamed tissues and organs, the closed circulatory system could therefore suffer a switch to acquire some of the characteristics of the open circulatory system and possibly characteristics of the hemolymph [114]. The inflammatory exudates bathe the cells of the injured tissues and organs thanks to the quick infiltration of the extracellular matrix [22]. Consequently, through the activation of the postcapillary venules and high endothelial venules endothelia, extravasation of plasma and blood cells is produced during the inflammatory response. An open pathway in the inflamed tissues and organs would therefore be established through the associated simultaneous activation of the lymphatic circulation [22,114]. Thanks to this new open microcirculatory system, where hemo-lymphatic circulation becomes predominant, tissues and organs are provided with molecules and cells coming from the upregulated extraembryonic coelomic-amniotic (i.e. neurogenic-related axis) and the trophoblast-yolk sac (i.e. bone-marrow-related axis) axes. Perhaps through the upregulation of ancestral phylogenetic mechanisms, the plasma, the interstitial fluid and lymph compartments could become closely linked during inflammation, thus favoring the continuous flow of fluid and cells from one compartment to the next [22,114] (Figure 1).

The venous endothelium acquires trophoblastic properties since it becomes an intermediary between the stressed host and the inflamed interstitium, playing the leading role in the host trophism [100,101]. The inflammatory venous endothelium, functioning like a trophoblastic barrier between the inflammatory interstitium and the injured organism, represents the site for the immunological recognition of damage and the pathogen-associated-molecular patterns (DAMPs and PAMPs) through the innate immune receptors, such as the Toll-like receptors (TLRs) and Nod-like receptors (NLRs) [117]. In this way, the inflammasome, a multi-protein complex including a NLR, is activated in response to PAMP and DAMP signals, which mediate the processing of intracellular pro-interleukin (IL)-1β and pro-IL-8 into their active secreted forms [117,118]. While DAMPs and PAMPs activate the inflammasomes, a variety of regulatory mechanisms minimize changes caused by the inflammatory activity, such as molecular chaperones and heat shock proteins [118].

Vertebrate cells contain cholesterol and although different tissues have characteristic patterns of a cholesterol metabolism, the basic pattern is similar in all cells [119]. During inflammation, cholesterol esters may be transferred from high density lipoproteins (HDL) to apolipoprotein E and apoliprotein B (apo B)-containing particles, such as low-density lipoproteins and very low density lipoproteins (LDL/VLDL). The trapping of apolipoproteins, particularly apo B containing particles, is an essential initiating event for the development of vascular inflammation [120]. Furthermore, the small non-coding RNA (microRNAs) could regulate the expression of key genes in cholesterol metabolism during inflammation [119]. Today, it is accepted that inflammation and the concomitant acute phase response induce marked changes in the lipoprotein profile [121] and cholesterol metabolism [122]. The characteristics of the inflammatory acute phase response and the associated cholesterol traffic have been compared with those playing the lead role in the accumulation of yolk materials into oocytes during oogenesis and their mobilization during embryogenesis [114].

The hypothesized endothelial inflammatory egg would turn into a reservoir of nutrients, including albumin. Bird embryos also consume egg white as a supplementary nutrient to yolk proteins [123]. Ovalbumin, ovotransferrin, trypsin inhibitor and other egg white proteins are absorbed into the extraembryonic cavity, through the albumen sac, then into the amniotic cavity and finally, reach the yolk sac through the vitello-intestinal duct [123].

Both cholesterol acquisition and leukocytic infiltration by the inflamed tissue collaborate to establish its new neuro-endocrine autonomy. Cholesterol is used locally to synthesize glucocorticoids and mineralocorticoids, which could regulate microcirculatory functions and immune cell activation. More evidence demonstrates the novo synthesis of glucocorticoids and mineralocorticoids in primary lymphoid organs, skin, brain and intestine [124]. Perhaps this ability of local corticosteroid synthesis is upregulated in the inflamed tissue due to the metabolic and functional needs of the neoformed tissue [114]. The pro-inflammatory and anti-inflammatory functions of androgens and estrogens and progesterone respectively, suggest that endogenous sex steroids may influence immune functions [125,126]. Progesterone, for example, increases vascular permeability and local accumulation of inflammatory cells. Clearly the role of progesterone in the inflammatory response merits further investigation [125]. In addition, leukocytes in the inflamed interstitium could develop neuroendocrine functions. Particularly, the pro-opiomelanocortin (POMC)-derived peptides found in leukocytes exhibit the same structure as those present in the pituitary [127]. Also, leukocyte-derived microparticles could harbor cytoplasmic proteins as well as bioactive lipids in the inflamed interstitium [128].

Inflamed tissues and organs acquire a progressive neuro-immune-endocrine autonomy through the previous recapitulation of extraembryonic functions. We have hypothesized that the phylogenetic evolution of our body is written in the successive phases making up the acute inflammatory response [129]. When the evolution of a severe traumatized patient is favorable, the patient could therefore undergo dedifferentiation followed by a process of differentiation (or specialization) representing a complete metamorphosis, just as it occurs with insects [128]. This specialization would require the return of oxidative metabolism and the development of a closed circulation, in which epithelial regeneration and angiogenesis are mainly involved, as well as the centralization of the neuro-immune-endocrine functions that are reduced to a symbolic presence in the periphery [22].

Angiogenesis allows for nutrition mediated by blood capillaries. The new functional properties of microcirculation include the exchange of oxygen, nutrients and waste products. This oxygen support induces oxidative metabolism, an efficient method for extracting energy from blood substrates. Oxygen and oxidative metabolism are an excellent combination through which cells can obtain an abundant energy supply for repairing tissues through specialized cells. This combination, however, produces energetic stress [12,25]. Perhaps the persistence in the adult microcirculation of a hypoxic venous territory, key for recapitulating those ancestral functions enabling development, is our safe-conduit to pass without vital risk through a hyperoxigenated environment. If so, then the maintenance of this microcirculatory hypoxic world in mammals has been made at the expense of either diverting oxygen through meta-arterioles or consuming and removing it through the specialized epithelia. In this case, our energetic way of life would be no less than a system for scavenging toxins with the ultimate aim of achieving the survival of our true pluripotential and ancestral gestation mechanisms. The return to ancestral phylogenetic mechanisms of life during surgical inflammation therefore opens a splendid path for translational research, as it has been previously shown in remodeling wound healing and inflammation in Drosophila [130].

The recapitulation of ontogenic and phylogenetic mechanisms during the inflammatory response in the mammal is an old hypothesis dating back to the 19th Century when Metchnikoff described macrophage functions [131]. He had already considered that the phagocytes were mediators of physiological inflammation, i.e. developmental conditions, ageing process and pathological inflammation. Therefore, Metchnikoff recognized a long phylogenetic and ontogenic history of the phagocytes. In fact, he suggested that the phagocyte functions were the same through evolution, but the context is what changed or was different [131].

Since then, many authors have posed the close similarity between the mechanisms of inflammation and other physiological processes, like animal regeneration [132-134] or the regulation of animal development [135]. If this were true, then we could consider the study of inflammatory mechanisms as a valuable tool for identifying the nature of countless processes of individual development as well as the phenotypic changes that occur during evolution [114,133].

## Conclusions

Surgical-related inflammation could be considered a high-grade stress response associated with an increasingly complex trophic functional system for using oxygen [131]. These mechanisms triggered by an acute stressor could be based on the recapitulation of ontogenic and phylogenic-related functions. Thus, the injured organism suffers processes of dedifferentiation, catabolism and autophagy mediated by the neuro-immune-response to stress. Consequently, the different types of endothelium that make up the microcirculation of the injured tissues and organs suffer a switch to turn into a functional endothelial inflammatory egg, capable of re-expressing extraembryonic functions to create a new tissue or organ.

It would be interesting to extrapolate this inflammatory response hypothesis to other types of inflammatory responses, as for example, the cause behind deep venous thrombosis. In the past, this was a common complication during the post-traumatic or post-operatory period [136], which today is prevented, to a great extent, using anticoagulant prophylaxis. In essence, the mechanisms that regulate its production are also developed at the endothelial level [137,138]. Thus, endothelial cells exposed to sustained hypoxemia or hypoxic injury could “cry for help” perhaps by developing a model of evolution that combines ischemia-reperfusion with hypercoagulability, leukocyte infiltration and remodeling by fibrosis [138]. The pathophysiological mechanisms that characterize the surgical inflammatory response could therefore be essential for the normal life of animals, such as invertebrates, far below mammals on the phylogenetic scale. In this paper, we have described those arguments that seem to demonstrate the existence of a recapitulation of ontogenic and phylogenetic mechanisms during the evolution of the surgical inflammatory response with an intensity related to the intensity and severity of the injury suffered by the body.

## Abbreviations

A: Aamnion; ACTH: Adrenocorticotrophin; BCE: Blood capillary endothelium; C: Capillary; CARS: Compensatory anti-inflammatory response syndrome; CRH: Corticotrophin-releasing hormone; D: Decidua; DAMPs: Damage- associated-molecular patterns; E: Epithelium; EM: Extraembryonic mesoderm; ESC: E endothelial precursor cell; EC: Exocoelomic cavity; HDL: High density lipoproteins; HEVE: High endothelial venule endothelium; HSCs: Hematopoietic stem cells; I: Interstitium; L: Lymphatic; LE: Lymphatic endothelium; Leu: Leukocytes; LDL: Low-density lipoproteins; M: Microbiome; MA: Meta-arteriole; MODS: Multiple organ dysfunction syndrome; MSCs: Mesenchymal stem cells; NE: Necrosed epithelium; NF-κB: Nuclear Factor-*kappa* B; NLRs: Nod-like receptors; PAMPs: Pathogen-associated-molecular patterns; PCVE: Postcapillary venule endothelium; POMC: Pro-opiomelanocortin; PTSD: Post-traumatic stress disorder; SC: Stem cell; SIRS: Systemic inflammatory response syndrome; T: Trophoblast; TLRs: Toll-like receptors; VLDL: Very low density lipoproteins; YS: Secondary yolk sac.

## Competing interests

The authors declare that they have no competing interests.

## Authors’ contributions

IP, CG and AA provided substantive editing and critical review. MAA, JIA, HY and JA wrote the manuscript. All authors read and approved the final manuscript.
